# Reducing HIV Risk among Transgender Women in Thailand: A Quasi-Experimental Evaluation of the Sisters Program

**DOI:** 10.1371/journal.pone.0077113

**Published:** 2013-10-29

**Authors:** Duangta Pawa, Rebecca Firestone, Sindh Ratchasi, Olivia Dowling, Yaowalak Jittakoat, Alex Duke, Gary Mundy

**Affiliations:** 1 Strategic Information Department, Population Services International/Thailand, Bangkok, Thailand; 2 Research and Metrics Department, Population Services International, Washington, D.C., United States of America; 3 Research and Metrics Department, Population Services International, Bangkok, Thailand; 4 Program Department, Population Services International/Thailand, Bangkok, Thailand; Alberta Provincial Laboratory for Public Health/University of Alberta, Canada

## Abstract

Transgender women are particularly at risk of HIV infection, but little evidence exists on effective HIV prevention strategies with this population. We evaluated whether Sisters, a peer-led program for transgender women, could reduce HIV risks in Pattaya, Thailand. The study used time-location sampling to recruit 308 transgender women in Pattaya into a behavioral survey in 2011. Coarsened exact matching was used to create statistically equivalent groups of program participants and non-participants, based on factors influencing likelihood of program participation. Using multivariable logistic regression, we estimated effects of any program participation and participation by delivery channel on: condom use at last sex; consistent condom and condom/water-based lubricant use in the past 3 months with commercial, casual, and regular partners; and receipt of HIV testing in the past 6 months. Program coverage reached 75% of the population. In a matched sub-sample (n = 238), participation in outreach was associated with consistent condom/water-based lubricant use with commercial partners (AOR 3.22, 95% CI 1.64–6.31). Attendance at the Sisters drop-in center was associated with receiving an HIV test (AOR 2.58, 95% CI 1.47–4.52). Dedicated transgender-friendly programs are effective at reducing HIV risks and require expansion to better serve this key population and improve HIV prevention strategies.

## Introduction

Thailand experiences a concentrated HIV epidemic with 1.2% of the general population, or 490,000, people estimated to be living with HIV [1]. While the Thai government has been credited with slowing the spread of HIV in the general population, key populations remain at significant risk for HIV infection [2], [3]. Transgender women represent a distinct population often subsumed within men who have sex with men (MSM), but with unique social, cultural and behavioral characteristics that make them particularly vulnerable to HIV infection [4].

A recent systematic review and meta-analysis found a high burden of HIV among transgender women globally and estimated HIV prevalence in Thailand at 12.5% [5]. Prevalence estimates from different studies within Thailand range from 10% to 17% [2], [6], [7]. Global epidemiological studies have placed HIV prevalence among transgender populations at anywhere from 8% to 68% [6], [8]–[10].

In Thailand, transgender women are a specific cultural grouping known in Thai as *kathoey*. However, this term is partially contested in the transgender community with some transgender women preferring to identify as “a second type of woman” (*sao praphet song*) [6], [11]–[15]. While transgender women may be discussed as a subcategory of MSM in the field of HIV prevention, transgender women in Thailand typically do not consider themselves MSM, in keeping with their feminine self-identity and in line with transgender populations in other settings across Asia, who frequently self-identify as a third gender [6]. Estimates of the transgender population in Thailand range from one in every 180 to 3000 persons, but exact population estimates are unknown and systematic, population-representative data is limited [16].

Transgender populations are at greater risk for HIV infection due to a variety of factors, including proximate factors such as multiple sexual partners and unprotected sex, both anal and neo-vaginal (vagina constructed during sex reassignment surgery). More distal factors such as social exclusion, experience of abuse, stigma, including self-stigma, and discrimination may interact with direct sexual behavior to influence HIV risks [5], [6], [17], [18]. Unprotected sex may occur with regular and non-regular partners, as 67.3% of transgender women in one recent Thai study reported consistent condom use during anal intercourse with casual partners, but only 39% consistent condom use with regular partners [6]. Among sex workers, 86% of participants reported engaging in unprotected anal sex with regular partners, and 27% reported unprotected anal sex with commercial partners [7]. Diverse sexual networks, including having both male and female partners, may facilitate HIV transmission [3], [19]–[21].

Although Thailand is perceived as generally tolerant towards its transgender population, the transgender community is politically disenfranchised and faces varying levels of stigma and discrimination [22], [23]. Transgender individuals are more likely to suffer from depression and lack support from family and friends [24]. Transgender women who use illicit drugs or have been abused by their father or brothers have been found to be less likely to use condoms during sex with commercial partners [7]. These social challenges, when paired with economic pressures, can exacerbate unsafe sexual behaviors among transgender women [7]. These trends are in line with other settings, where transgender women face elevated mental health risks [25].

A handful of studies outside of Thailand have evaluated behavioral HIV prevention interventions for transgender women [26]–[28]. These suggest that targeted interventions tailored to the needs of this population can contribute to HIV risk reductions, particularly if they are delivered through an approach that addresses other transgender health concerns, such as hormone use, gender re-assignment surgery, etc., and incorporate efforts at stigma reduction. Despite ample evidence of the relative vulnerability of transgender women, we were not able to identify any studies from Thailand that systematically evaluated a HIV prevention intervention for transgender women.

### Sisters: HIV prevention for transgender women in Pattaya, Thailand

Since 2004 Population Services International (PSI) has implemented the Sisters program to prevent HIV among transgender women in the city of Pattaya in eastern Thailand. Pattaya is a center for transgender activity, with employment opportunities for transgender women in the entertainment, hospitality, tourism, and commercial sex sectors. Social exclusion tends to limit employment opportunities for transgender women in Thailand and other settings, with commercial sex becoming one of the most viable options for transgender women to support themselves and their families [18]. As a hub for the tourism industry, Pattaya has become a common destination for transgender women migrating for work, and commercial sex - either part or full time - is often one of the primary options available [29].

Sisters was designed by gathering extensive insight into Pattaya's transgender community and uses a social marketing approach to address key motivators and barriers to HIV risk reduction [30]. With the stigma that transgender women face in Thai society, Sisters addresses HIV prevention within the context of providing a safe haven and broader social support to transgender women. Staff, peer educators, and volunteers are all transgender women.

The program operates through three service delivery channels. Sisters offers a drop-in center (DiC) that provides counseling, social services, and on-site HIV and STI testing. In partnership with a local public hospital, a transgender nurse has provided HIV testing and counseling (HTC) at the drop-in center for more than six years. Transgender women who tested for HIV returned 3–7 days later to receive results. After experiencing substantial loss to follow-up with this model, Sisters became a pilot site in September 2011 for a Ministry of Public Health supported initiative to provide rapid HIV testing services, with pre- and post-test counseling offered within one hour [30].

Sisters also engages in peer-led interpersonal communication. Outreach workers operate in transgender bars, clubs, and parks to reach transgender women with messages on safe sexual behaviors and HTC. Outreach workers target areas where commercial sex is available, provided by freelance transgender sex workers or through transgender entertainment/hospitality workers who may also participate in commercial sex. Outreach workers also seek to reach transgender women involved in other occupations. Outreach workers promote awareness of Sisters and its DiC services, and they distribute condoms and water-based lubricants [30].

More in-depth communications are provided through home visits. Peer educators meet clients in a safe, private location for counseling, psychosocial/emotional support, and information on gender reassignment, hormone therapy, and cosmetic surgery. Peer educators facilitate linkages to transgender-friendly government health services and will accompany Sisters clients to appointments for STI treatment, CD4 tests, and anti-retroviral treatment.

We aimed in this study to assess the effectiveness of Sister's model for reducing HIV risks among the transgender population in Thailand. We looked for evidence that participation in Sisters is related to having received an HIV test and to using condoms and water-based lubricant consistently. Because there is evidence that transgender women have a range of sexual partners, we examined condom and water-based lubricant use for commercial, non-commercial regular, and non-commercial casual partners.

## Materials and Methods

### Location and sampling strategy

We used data from a cross-sectional survey of the transgender population in Pattaya, Thailand. Data was collected in December 2011 using time-location sampling (TLS). TLS is a location-based sampling technique used when the study population congregates in certain venues such as bars, parks, or street corners in red light districts [31]. TLS has proven effective in reaching hard-to-reach populations by directly targeting respondents at the sites they frequent in order to obtain a representative sample of the population [32]. The research team surveyed sites of transgender activity in Pattaya through ethnographic mapping; the list of sites was then used as a sampling frame to select a probability sample of sites. Four locations were selected where transgender people congregate from 8 PM to midnight for two weeks in the month of December. The research team worked with interviewers who came from the target population to reduce the risk of response biases. All transgender women at study locations during the sampling time period were approached by the study team. Peer educators from Sisters assisted in screening potential respondents. Interviews were then conducted in a private and conveniently located setting, with only the interviewer and potential respondent present.

### Study population

Inclusion criteria for study participation were as follows: respondent self-identified as transgender and expressed sexual attraction to the masculine gender; respondent had had any type of penetrative (anal, vaginal or neovaginal) sex with more than one sexual partner in the past three months; respondent was resident in Pattaya for more than one month, and could read and speak Thai. Potential respondents were excluded from the study if they were incapacitated due to drug or alcohol use and not able to complete an interview within two hours of being sampled. All potential respondents meeting the screening criteria were then asked to consent to participate. During data collection, 336 individuals were screened. Of these, 5 individuals were not eligible for participation, and 23 individuals refused to participate. A structured questionnaire was used to collect data and administered to respondents by interviewers. The questionnaire included modules on socio-demographic characteristics, sexual behaviors, HIV and STI testing, and participation in Sisters program activities. A qualitative study using in-depth interviews with 14 transgender individuals in September 2011 was used to inform questionnaire development, and the questionnaire was then pre-tested [33].

### Ethical considerations

Data collection was completely anonymous, and no identifying information was collected from respondents. Program staff advised the study team that transgender people in Pattaya would be unwilling to provide signatures or written consent due to concerns about stigma and safety of identifying information. An informed consent script was read to the respondent by the interviewer, and the interviewer indicated consent on the questionnaire. The PSI Research Ethics Board reviewed and approved the survey protocol and this consent procedure.

### Outcome measures

Four outcomes were used for this analysis: 1) Condom use at last sex, 2) consistent condom use in the past three months, 3) consistent condom and water-based lubricant use in the past three months, and 4) receipt of an HIV test in the past six months. Consistency in condom and condom/water-based lubricant use was defined as using a condom or condom with water-based lubricant from start to finish for each sex act with a specified partner type for the defined recall period. We measured condom and water-based lubricant use by partner type for commercial, casual, and regular partners. Commercial partners were defined as partners to whom the respondent had given or received money or other valuables in exchange for sex. Casual partners were defined as partners with whom respondents engaged in sexual activity with no reported emotional commitment and had not given or received money or valuables in exchange for sex. Regular partners were defined as partners with whom respondents engaged in sexual activity with an emotional commitment or sense of attachment.

### Program participation

All measures of program participation were constructed as dichotomous variables. Participation in Sisters was measured as receipt of any Sisters services in the past 12 months. We assessed specific channels of program participations as follows: attendance at the Sisters drop-in center, interaction with a Sisters outreach worker (at workplace, entertainment venue, or event), or home visit from a Sisters peer educator.

### Other measures

We also considered several other measures. Socio-demographic variables included age, education, duration of residence in Pattaya, occupation (categorized by likelihood of involvement in commercial sex), monthly income, and number of transgender friends. Sexual behaviors and practices included total and type of sexual partner in the past year (commercial, causal, regular), whether the respondent practiced penetrative and/or receptive sex (anal, vaginal or neo-vaginal), and whether the respondent had had sex while drunk or high on drugs/alcohol in the past 3 months.

### Analysis approach

To improve causal inferences of estimates of program effectiveness, we used statistical matching as a quasi-experimental method to enable clear designation of a counterfactual [34]. An experimental design was not an option with on-going program implementation and with a hard-to-reach population. We applied coarsened exact matching to create a sample of treated cases (program participants) and control cases (non-participants) from observational data, matched on observed factors that influenced selection into treatment [35], [36]. Coarsened exact matching (CEM) is a monotonic imbalance matching method designed to reduce imbalance between treatment and control groups derived from observational data [37]. CEM decreases model dependence and the need for iterations in balance checking and re-matching, and the technique yields estimates with lower variance and bias at the same sample size [36], [38], [39]. CEM assigns each case into one of a specified set of strata in which members are exactly matched on a set of coarsened, i.e. categorized, variables. Matched members are then assigned a weight specific to that stratum and representative of the proportion of all members present in the stratum.

Using coarsened exact matching, we matched respondents in the study sample on factors likely to influence program participation. Matching variables were identified through consultation with program managers. Identification of variables focused on transgender women's involvement in commercial sex activities, which would influence likelihood of being reached by an outreach worker, plus factors reflecting likelihood of participating in the transgender community in Pattaya, which would influence opportunities to attend the drop-in center. These variables included duration of residence in Pattaya (less than one year/more than one year), monthly income (continuous variable coarsened with a cutpoint of <20,000THB/month and ≥20,000THB/month, US$1 = 30THB), working in an entertainment venue, working as a freelance sex worker, and number of transgender friends (coarsened to 0–4, 5–7, 8–18, 19–320 according to how data in the sample was distributed), as a measure of the density of transgender social networks. We assessed relationships between potential matching variables and any program participation using Spearman correlation coefficients, which ranged from r = 0.067 to r = 0.193, and we used all of these variables in the matching procedure. An exact match made on the coarsened variables yielded an L1 measure of 1.735E^−17^, indicating minimal imbalance between program participants and non-participants [37].

We derived descriptive statistics for the full sample and matched sub-sample. We then used multivariable logistic regression to estimate odds ratios of average treatment effects for each measure of program participation. We first estimated unadjusted odds ratios for each outcome of interest. We then adjusted for different channels if one channel was statistically significant, to determine whether the channels operated independently of each other. We tested interaction effects of DIC*outreach and DIC*home visit. Results were not statistically significant and not shown. We used the same modeling approach in the full sample and with the matched sub-sample to estimate how causal inferences changed following matching. All analyses were conducted in Stata 10.1.

## Results

In total, 308 transgender women participated in this study. Table 1 presents characteristics of transgender survey respondents. Mean age was 24.6 years. Most transgender women in our study had obtained a high school education or higher, and about half of the participants (53%) had lived in Pattaya less than one year. Sixty percent of transgender women in the survey were employed in evening entertainment venues, and the largest percentage of respondents (37%) had a monthly income between 10,000–20,000 Thai baht (US$333–666), followed by 20,001–30,000 baht (US$667–1000). Respondents had extensive transgender friend networks (mean number of transgender friends = 14), despite being recently resident in Pattaya.

**Table 1:** Population characteristics by sample.

	Full sample (n = 308),%	Matched sample (n = 238),%
**Socio-demographic characteristics**		
***Age, mean(sd)***	24.56 (0.23)	24.55 (0.27)
***Education***		
Primary school and lower	6.17	7.23
Secondary school	39.29	43.49
High school	43.18	39.21
Diploma and higher	11.36	10.07
***Duration of residence in Pattaya***		
Years, mean (sd)	2.59 (0.22)	2.65 (0.25)
≤ one year, %	52.92	48.92
> one year, %	47.08	51.08
***Occupation***		
Employment in evening entertainment venue	60.06	59.16
Freelance sex worker	39.29	42.00
Other employment	8.12	5.55
***Monthly income***		
0–10,000 THB	19.81	15.00
10,001–20,000 THB	36.69	36.08
20,001–30,000 THB	25.32	26.48
>30,000 THB	18.18	22.44
***Transgender friends, mean (sd)***	14.28 (1.29)	17.29 (1.67)
**Sexual behaviors and practices**		
***Sexual partners in the past 12 months***		
Mean number of partners, any type (sd)	41.21 (2.82)	44.01 (3.49)
*Commercial partners*		
%	93.83	97.90
Mean number of partners (sd)	38.63 (2.84)	39.47 (3.41)
*Casual partners*		
%	13.96	12.18
Mean number of partners (sd)	8.86 (1.44)	9.43 (1.91)
*Regular partners*		
%	17.86	13.45
Mean number of partners (sd)	1.15 (0.054)	1.11 (0.06)
***Sexual role***		
Always practice receptive sex	38.31	41.08
Always practice penetrative sex	0.97	1.84
Practice both receptive and penetrative sex	60.71	57.08
***Had sex after using drugs or alcohol in past 3 months***	52.27	51.09

In terms of sexual behaviors, respondents had large sexual networks, with mean number of sex partners in the past year at 41. The majority of these partners appeared to be commercial in nature, as over 90% of respondents reported commercial partners in the past 3 months. A comparatively smaller percentage of respondents reported having casual (14%) or regular (18%) partners. Just under two-thirds of participants (61%) reported engaging in both receptive and penetrative sex, and 38% reported being the receptive partner during every sex act. Around half of respondents (52%) reported having sex while drunk or high in the past 3 months.

Table 2 shows outcomes of interest and program participation. Condom use at last sex was widespread (93%). A trend was present in condom and water-based lubricant use by partner type in the past 3 months. Condoms were used with greater consistency than condoms with water-based lubricant, and both methods of protected sex were more common with commercial partners, dropping off with casual and then regular partners. This trend was present in the full and matched samples. In the full sample, 85% of respondents with commercial partners reported using condoms consistently while 49% of respondents with regular partners reported using condoms and water-based lubricant consistent. Out of the total sample, 54% of respondents reported receiving an HIV test in the last six months.

**Table 2:** Behavioral outcomes and program participation, by sample.

	Full sample (n = 308),%	Matched sample (n = 238),%
**Behavioral outcomes**		
Condom use at last sex with any partner	93.18	92.44
***Commercial partners***		
Consistent condom use in the past 3 months	85.81	84.77
Consistent condom and water-based lubricant use in past 3 months	75.09	72.72
***Casual partners***		
Consistent condom use in the past 3 months	72.09	61.75
Consistent condom/water-based lubricant use in the past 3 months	62.79	55.44
***Regular partners***		
Consistent condom use in the past 3 months	60.00	61.73
Consistent condom/water-based lubricant use in the past 3 months	49.09	36.32
***HIV testing***		
Received an HIV test in the past 6 months	53.90	54.68
**Program participation in the past 12 months**		
Received any Sisters service	75.65	72.31
Visited Sisters drop-in center	39.61	39.92
Received Sisters outreach	67.86	64.29
Received Sisters home visit	17.53	16.81

Sisters program coverage was also widespread (Table 2). Three-quarters of transgender women (76%) had received any Sisters services in the past 12 months. Most of this participation was likely through outreach contacts (68%), as only 40% of survey respondents attended the drop-in center, and 18% received a home visit.

In Table 3 we present estimates of program effects from logistic regression models of the full and matched samples. In the matched sample, transgender women who participated in any Sisters services in the last 12 months were more likely to use a condom at last sex with any partner (OR 3.75, 95% CI 1.41–9.97) as a bivariate relationship. Transgender women who participated in any part of the program were also more likely to use condoms and water-based lubricant consistently with commercial partners (OR 2.37, 95% CI 1.28–4.41). No statistically significant relationships were found between program participation and condom or condom/water-based lubricant use with casual or regular partners.

**Table 3:** Logistic regression estimates of program participation associations with condom use, condom/lubricant use, and HIV testing in matched and unmatched samples of transgender women in Pattaya, Thailand, 2011*.

		Full sample (n = 308)						Matched sample (n = 238)					
				Model 1		Model 2				Model 1		Model 2	
		OR, 95% CI	p-value	Adjusted OR, 95% CI	p-value	Adjusted OR, 95% CI	p-value	OR, 95% CI	p-value	Adjusted OR, 95% CI	p-value	Adjusted OR, 95% CI	p-value
**Condom use at last sex, any partner**													
Any service	2.51 (1.01–6.22)		0.047					3.75(1.41–9.97)	0.008				
Drop-in center	1.70 (0.64–4.50)		0.289	1.48 (0.54–4.02)	0.445			2.48 (0.79–7.74)	0.121	1.92 (0.59–6.24)	0.280		
Outreach	2.02 (0.83–4.94)		0.122	1.86 (0.75–4.65)	0.182			3.10 (1.15–8.32)	0.025	2.68 (0.97–7.40)	0.057		
Home visit	4.53 (0.59–34.50)		0.145					3.66 (0.47–28.34)	0.214				
***Commercial partner (full sample n = 289, matched sample n = 233)***													
**Consistent condom use in the past 3 months**													
Any service		1.06(0.49–2.28)	0.888					1.58 (0.74–3.38)	0.238				
Drop-in center		1.06 (0.54–2.08)	0.875					1.36 (0.64–2.88)	0.422				
Outreach		0.90 (0.44–1.86)	0.780					1.30 (0.63–2.70)	0.483				
Home visit		0.91 (0.40–2.11)	0.834					0.82 (0.33–2.02)	0.661				
**Consistent condom/water-based lubricant use in past 3 months**													
Any service		1.23 (0.67–2.27)	0.510					2.37 (1.28–4.41)	0.006				
Drop-in center		0.79 (0.46–1.35)	0.390	0.70 (0.40–1.22)	0.210	0.70 (0.40–1.22)	0.211	1.03 (0.57–1.86)	0.912	0.79 (0.42–1.48)	0.460	0.80 (0.43–1.51)	0.490
Outreach		1.59 (0.91–2.78)	0.103	1.73 (0.97–3.09)	0.062	1.75 (0.95–3.21)	0.074	2.72 (1.50–4.92)	0.001	2.89 (1.56–5.37)	0.001	3.22 (1.64–6.31)	0.001
Home visit		1.16 (0.57–2.36)	0.672			0.98 (0.46–2.08)	0.951	1.15 (0.53–2.52)	0.723			0.68 (0.29–1.62)	0.381
***Casual partner (full sample n = 43, matched sample n = 29)***													
**Consistent condom and water-based lubricant use in the past 3 months**													
Any service		0.81(0.17–3.80)	0.787					0.49 (0.03–7.28)	0.604				
***Regular partner (full sample n = 55, matched sample n = 32***													
**Consistent condom use in the past 3 months**													
Any service		1.00(0.30–3.36)	1.000					0.35 (0.06–1.91)	0.225				
**Consistent condom and water-based lubricant use in the past 3 months**													
Any service		1.14(0.35–3.75)	0.826					1.95 (0.38–10.08)	0.79				
**Received an HIV test in the past 6 months**													
Any service		3.32(1.90–5.76)	0.000					2.45 (1.36–4.39)	0.003				
Drop-in center		3.17(1.95–5.14)	0.000	2.84 (1.73–4.66)	0.000	2.83 (1.72–4.65)	0.000	2.80 (1.62–4.83)	0.000	2.60 (1.48–4.56)	0.001	2.58 (1.47–4.52)	0.001
Outreach		2.24 (1.37–3.65)	0.001	1.84 (1.11–3.06)	0.018	1.64 (0.96–2.80)	0.068	1.72 (1.01–2.93)	0.047	1.38 (0.79–2.41)	0.263	1.29 (0.72–2.34)	0.392
Home visit		2.11(1.13–3.94)	0.019			1.59 (0.81–3.13)	0.179	1.67 (0.83–3.39)	0.153			1.29 (0.60–2.78)	0.518

* Multiple logistic regression models only estimated when factors statistically significant at p<0.05 in bivariate models.

Breaking out program participation by channel, participation in outreach was associated with condom at last sex (OR 3.10, 95% CI 1.15–8.32) as a bivariate relationship. This relationship was attenuated and borderline statistically significant after adjusting for drop-in center participation (AOR 2.68, 95% CI 0.97–7.40). Outreach was associated with consistent use of condoms and water-based lubricant with commercial partners in a bivariate model and then after adjusting for drop-in center and home visit participation (AOR 3.22, 95% CI 1.64–6.31).

For HIV testing, a statistically significant relationship was found with program participation (OR 2.45, 95% CI 1.36–4.39). Disaggregating by channel, participation in activities at the Sisters DiC was associated with transgender women respondents having received a test in the past 6 months (AOR 2.80, 95% CI 1.62–4.83).

## Discussion

We aimed to assess whether a targeted HIV prevention program for transgender women in Pattaya, Thailand could reduce HIV risks through promotion of condom and water-based lubricant use and HIV counseling and testing, and we find positive evidence to that effect.

Sisters was effectively able to reach large portions of transgender women in Pattaya, despite the transient nature of this population. Condom use in this population was high, and HIV testing was relatively common. Transgender women had multiple sex partners, the majority of whom were commercial partners, and three-quarters of women used condoms and water-based lubricant consistently with these partners.

We found that the program influenced condom use at last sex. When disaggregating by channel, program outreach was associated with condom and water-based lubricant use with commercial partners, but other channels were not. This relationship was not apparent in the full sample but was in the matched sample, as effect estimates tended to be larger in the matched sample. We found no evidence that the program had an influence on condom and lubricant use with casual or regular partners. This is likely an artifact of small sample size, since very few respondents reported these partner types.

We suspect that null results for consistent condom use alone with commercial sex partners, partnered with a positive result for condom and water-based lubricant use are explained by program activities to make water-based lubricants more acceptable and accessible. Condom use is generally high among transgender women in Thailand, particularly with commercial sex partners [6], [40]. In this context, Sisters has organized communications to raise awareness about the benefits of water-based lubricant and to discourage use of oil-based and other lubricants that can damage condoms. A combined package of condom and water-based lubricant was also introduced into outreach activities, replacing condom-only distribution.

Evidence suggests that this approach is working. Sisters has achieved generally high levels of outreach coverage. With a transient population, it is important for the program to maintain coverage and frequency of outreach contacts. The outreach interaction itself – short messages plus condom and water-based lubricant distribution – is likely sufficient for this population with high levels of knowledge on condom use and HIV risks and motivated to use HIV prevention methods based on factors such as self-efficacy and positive attitudes [40].

Receipt of an HIV test was strongly related to participation in Sisters services. In the matched sample, Sisters participants were two and a half times more likely to have received in HIV test than those who had not participated. Participation in the DiC was likely driving this relationship, as the effects of outreach dropped out in adjusted models.

We suspected that the DiC effect on HIV testing was attributable to rapid HIV testing introduced in September 2011. We triangulated our results against program records on HIV testing and counseling sessions conducted before and after the initiation of rapid testing, and we looked at the monthly testing caseload covering the study recall period. Thirty-two cases received HIV testing at the drop-in center from June–August 2011, while 134 cases received an HIV test from September–December 2011.

These findings suggest that rapid testing at the DiC is an effective mechanism to promote HTC in this population. At the very least, rapid HIV testing should be institutionalized at the DiC, and opportunities for expansion of this service should be identified, in coordination with provincial health authorities. Because only 40% of the population had attended the DiC, Sisters should consider promotional communication and additional outreach strategies to drive more people to the drop-in center. Alternately, the program could consider how the model developed in the DiC can be adapted to other health and social services that transgender women frequent, such as placing Sisters trained peer educators inside government health services or developing guidelines for specifying services as transgender-friendly.

Our efforts to contextualize the results of this study are hampered by minimal published data on evaluations of HIV prevention interventions for transgender women. Available evidence suggests that our findings are in line with other studies showing that targeted outreach efforts through community based initiatives, peer education, support groups and counseling can increase positive perceptions of condom use, self-efficacy in negotiating condom use, greater self-esteem, and a decrease in sexual risk behaviors including fewer sexual partners [28], [41]–[44]. A survey of transgender women in Laos exposed to a transgender-targeted social marketing intervention registered higher levels of condom use at last anal sex with casual partners and greater use of water-based lubricant [26]. Attitudes towards consistent condom use also improved along with condoms use with regular partners. MSM and transgender networks in Bangkok have also been shown to link vulnerable transgender women to essential health services, including HIV testing and counseling [45].

The retrospective nature of this study presents several limitations. Recall bias in the outcomes, particularly on consistent condom and lubricant use, is possible, although levels of condom use were comparable to previous rounds of data collection [40]. Respondents may have under-reported program participation, potentially attenuating estimates of program effects. Transgender women who have non-commercial partners may have been under-sampled in the application of time-location sampling. This does not influence inferences on estimates of program effects regarding commercial partners but suggests that we had limited power to assess program effects for casual and regular partners. Respondent-driven sampling may be more effective for reaching these individuals but is more resource-intensive.

Another potential limitation was in the coarsening of variables before matching. The matching procedure we used faces a trade-off in increasing the number of matched pairs at the expense of less exact matching. Categorical variables, including two occupation variables and duration of residence in Pattaya, were not affected by this potential source of bias. Income was coarsened at the median, and number of transgender friends was coarsened according to meaningful groups that reflected how the variable was distributed. Differences in transgender women within these groups are thought to be less programmatically important in terms of the density of social networks than difference between categories. As with other matching procedures, our analysis using coarsened exact matching is also at risk for omitted variable bias in that we cannot make inferences about the effect of unobservables on participation in Sisters services and subsequent outcomes.

### Conclusion

Our findings suggested that an HIV prevention program targeted to transgender women can address HIV-related risks, evidence that is needed given the substantial HIV burden this population faces in Thailand and globally [5]. Key elements appear to be making water-based lubricant accessible along with condoms in outreach activities, and embedding rapid HIV testing in community-based, transgender-friendly services.

Despite having evidence on HIV risk reduction through HTC and condom/water-based lubricant use – the aims of this study – we do not know whether Sisters is able to achieve risk reduction or health promotion in other areas of transgender health. Transgender women as a marginalized population have other health risks, particularly around violence, substance use, and mental health. Additional research is needed to determine whether Sisters can achieve a comprehensive approach to transgender health and social services.

These findings also highlight the potential value of developing more broad-based HIV prevention programming specific to transgender women and independent of activities for MSM in Thailand. This response could include dedicated sampling of transgender women in integrated bio-behavioral surveys of HIV prevalence plus identification of transgender women as a specified key population in government plans for HIVAIDS control and in reporting to the United Nations. These efforts would contribute to more effectively meeting the needs of this key population.
